# The safety of a novel single-drape cover for sterile back tables in the operating room compared to the standard two-drape method: an experimental study

**DOI:** 10.1186/s13037-022-00330-z

**Published:** 2022-06-02

**Authors:** Mohammadreza Zarei, Saeed Babajani-Vafsi, Mohammad Hassan Kazemi-Galougahi, Ashraf Bakhshi, Neda Mirbagher Ajorpaz, Mahdi Ghorbani

**Affiliations:** 1 Department of Surgical Technology, Faculty of Paramedical Sciences, Aja University of medical sciences, Tehran, Iran; 2 Department of Social Medicine, Faculty of Medicine, Aja University of Medical Sciences, Tehran, Iran; 3 Department of Microbiology, Qazvin University of Medical Sciences, Qazvin, Iran; 4 Autoimmune Diseases Research Center, Kashan University of Medical Sciences, Kashan, Iran; 5 Department of Medical Laboratory sciences, Aja University of Medical Sciences, Tehran, Iran

**Keywords:** Operating rooms, Infection control, Perioperative care, Surgical site infection, Surgical instruments

## Abstract

**Background:**

Covering the prepared sterile back tables (PSBTs) during periods of nonuse and during active surgeries may decrease contamination of sterile surgical instruments that have direct contact to surgical wound. The Association of periOperative Registered Nurses (AORN) declared that an easy method for covering and removing the drape will ultimately be most effective (e.g. standard two-drape method). Hence, this study was designed to test the hypothesis that using a novel single-drape cover had more efficiency and safety in decreasing airborne bacteria-carrying particles (ABCPs) settling on the PSBTs during static and dynamic periods than the standard two-drape method.

**Methods:**

This experimental study was conducted with using 918 agar plates to detect contamination of the PSBTs with ABCPs on two conditions (static and dynamic) at an academic medical center in Kashan, Iran, from September 25, 2021, to January 20, 2022. The contamination of PSBTs was evaluated by 6 agar settle plates (*n* = 918 in total) on each PSBT in static and dynamic operating room (OR) conditions. At each time-point, this set-up was repeated on two occasions else during data collection, establishing 81 PSBTs in total. Tested groups included the PSBTs covered with the standard two-drape method, the novel single-drape cover, or no cover. The plates were collected after 15, 30, 45, 60, 120, 180, 240 min and 24 h. The primary outcome measured was comparison of mean bioburden of ABCPs settling on covered PSBTs on two conditions by using agar settle plates. The secondary outcomes measured were to determine the role of covering in decreasing contamination of PSBTs and the estimation of time-dependent surgical instrument contamination in the uncovered PSBTs on two conditions by using agar settle plates.

**Results:**

Covering the PSBTs during static and dynamic OR conditions lead to a significantly decreased bioburden of ABCPs on them (*P* < 0.05). No differences were seen between the standard two-drape method and the novel single-drape cover (*P* > 0.05).

**Conclusions:**

We found that there is no preference for using the novel single-drape cover than the standard two-drape method. Our results showed a significant decrease in bioburden of ABCPs on the PSBTs when those were covered during static and dynamic OR conditions, indicating the efficiency for covering the PSBTs during periods of nonuse and during active surgery.

## Background

Surgical site infections (SSIs) are the most significant postoperative complication. SSI is associated with increased morbidity and mortality, additional therapeutic interventions and increased healthcare system and patients costs [1]. The sources of microorganisms that cause SSIs in healthcare facilities are multifactorial in origin and may be endogenous (e.g. patient’s own normal skin flora) or exogenous (e.g. airborne particles [APs]) [2, 3]. The skin flora of patients is the direct source of contamination in only 2% of cases, leaving 98% of cases connected to APs [4].

Previous studies showed 80 to 90% of pathogenic bacteria isolated of surgical wounds have correlated with APs in operating rooms (ORs), with [5] airborne skin scales (flakes) acting as vectors (carriers) for pathogenic bacteria that infect surgical wounds [6].

It is accepted that most ORs are not free from airborne bacteria-carrying particles (ABCPs) [7] and during open surgery, surgical wound contamination by ABCPs may occur in 30% of cases to directly falling down of the ABCPs on the surgical wound and in 70% of cases to settling on the surgeon’s hands and surgical instruments and then be transferred indirectly into the surgical wound [8, 9].

Surgical wound contamination by ABCPs plays a key role in the exogenous pathogenesis of SSIs. Hence, contamination with ABCPs in ORs should be minimized and controlled to protect patients [10]. OR nurses as a member of the surgical team are responsible for decreasing the environmental risks of SSI with implementing hygiene and aseptic principles in the OR [11].

Thus, finding ways to diminish SSIs is of utmost importance by them and other specialists in the team, both for patient’s safety and for optimal resource utilization within healthcare facilities [11, 12].

One of cost-effective ways may be covering the prepared sterile back tables (PSBTs) during periods of nonuse. The practice of covering may be used when an operation will be delayed. This would allow the PSBTs to be protected until the surgical procedure can commence [12].

However, prior to 2013, the Association of Perioperative Registered Nurses (AORN) advised against covering or draping PSBTs because it was believed that the cover could not be removed without contaminating them and it did not support covering the PSBTs with any type of cover [12, 13].

On the other hand, the most recent update from the Association of Surgical Technologists (AST) still does not approve the use of back table covers. They, in their statements “Standards of Practice for Creating the Sterile Field”, declared to aseptically remove the cover that prevents contamination of the PSBTs cannot be achieved. Because, the parts of the cover are below the level of the surface of the PSBTs and most likely will touch the sterile surfaces of back table onto removal [14]. The rationale for this ideology originates from the theory that bringing the part of the cover that was below the PSBTs above sterile table may allow air currents to draw microorganisms and other contaminants (e.g., debris, dust) from the floor and deposit them in the PSBTs [12, 14]. However, their theory is not supported by any evidence [14]. There was no evidence to show removal of the cover contaminates the PSBTs until, in 2013 based on two studies [15, 16], the AORN changed their recommendations to suggest covering PSBTs during prolonged periods of nonuse.

The AORN recommends using a two-drape method instead of a single-drape one, while it has not published any evidence about the preference of the two-drape method compared to the single-drape one. Hence, additional evidence to support or refute the single-drape method should be performed. Previous studies showed the single-drape method with one sterile drape is benefit when used to cover PSBTs [12, 15, 17], but it was not applied per the AORN recommendations. The AORN currently recommends perioperative team members should use a two-drape method (i.e., to cover the PSBTs by using two sterile cuffed drapes) that allows the cover to be applied and removed without contamination of the PSBTs [12, 13].

However, AORN has not provided any published evidences for this preference why a two-drape method is safer than a single-drape method. *Wicklin* declared that further research is required to select best methods for covering PSBTs [13]. The evidence showed covering PSBTs in a method that prevents contamination on removal is a practice that should be used by OR nurses [12, 13].

According to the ideology [12, 14] and due to a lack of evidence on safety of the standard two-drape method regarding to decrease contamination PSBTs with ABCPs, we decided to design and construct the novel single-drape cover that may be removed from PSBTs without contaminating them and then compared it with the recommended standard two-drape method by the AORN. Also, they states “The health care organization should develop a standardized procedure in collaboration with infection prevention personnel for covering sterile fields to delineate the specific circumstances when sterile fields may be covered and to specify the method of covering and the length of time a sterile field may be covered” [12, 13]. The AORN declared that an easy method for covering and removing the drape will ultimately be most effective. Hence, this study was designed to test the hypothesis that using the novel single-drape cover had more efficiency and safety in decreasing ABCPs settling on the PSBTs during static and dynamic periods than the standard two-drape method. We were layout an OR environment to evaluate the degree of airborne bacterial contamination on the PSBTs during both static (without surgical activity or at rest) and dynamic (during simulated surgery) environments.

## Methods

### Study design and sample

This experimental study was conducted with using 918 agar plates to detect contamination of the PSBTs with ABCPs on two conditions (static and dynamic) at an academic medical center in Kashan, Iran from September 25, 2021, to January 20, 2022.

According to the ethics committee authority of Aja University of Medical Science, receiving ethics approval was not necessary for this study. The issues relating to health laws, safety of patients, and ethical considerations resulted in that we did not perform these experiments during real surgeries. Hence, no patients were included in our study and it was conducted in condition of the mock surgery procedure instead of a real surgery (project number IR.AJAUMS.REC.1400.018). All static and dynamic tests were performed at surgical department within one hospital. The OR had a turbulent ventilation system (with 4 array diffusers in the ceiling and 2 return grilles, air inflow of ~ 600 L/h, air recirculation ~ 15 air changes/hour). During data collection periods, the OR had a means temperature of 24 °C (SD: 0.77) during static testing and 23.8 °C (SD: 0.70) during dynamic testing, and the air humidity ranged from 38 to 55% with means of 50.97% (SD: 5.2) during static testing and air humidity ranged from 45 to 55% during dynamic testing 51.64% (SD: 4.1).

**Fig. 1:**
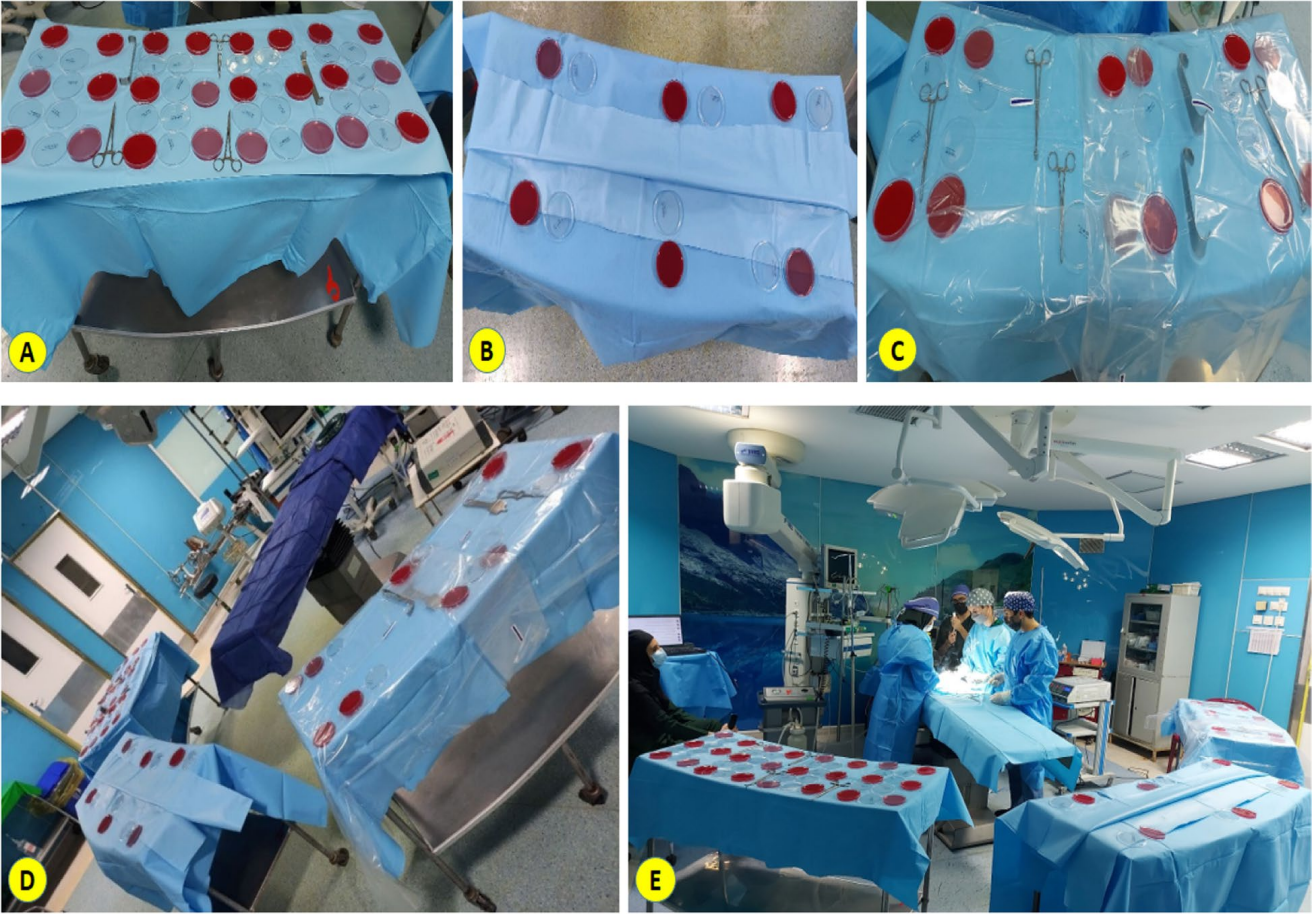
The prepared sterile back table cover setup: experiments with (**A**) no cover (Group1), (**B**) the standard two-drape method (Group2), and (**C**) the novel single-drape cover were undertaken to determine the effectiveness of decreasing the ABCPs beneath compared to top cover; OR Layout for mock surgery experimental setup: (**D**) during dynamic testing and (**E**) static testing

A total of 918 agar plates were used to detect airborne bacterial contamination of PSBTs, comprising 612 5% *Sheep blood agar plates* (*Merck KGaA, Darmstadt, Germany*) for G (+) bacteria and 306 *MacConkey* agar plates (*Merck KGaA, Darmstadt, Germany*) for G (−) bacteria. The study consisted 81 back Tables (81 total back tables with 3 back tables tested at each time point) and 30 sterile instrument trays that were aseptically opened and then few instrument put on 3 separate prepared back tables (one sterile instrument tray was opened per 3 back tables at each time point) in the OR. Then, they were set immediately with 3 different ways:

1. Group 1 (no cover; as a control) (Fig. 1-A).
2. Group 2 (the standard two-drape method; using two disposable nonwoven sterile drapes) (Fig. 1-B).
3. Group 3 (the novel single-drape cover with medical grade polyethylene texture) (Fig. 1-C).

### The novel single-drape cover

We with cooperation personnel of hospital infection control designed a cover that could possibility be removed without contamination of PSBTs and assessed its effectiveness for preventing airborne bacterial contamination in preoperative and intraoperative. Also, the novel single-drape cover is cost-effective and can be put on any surgical wound in cases surgical team are waiting for results of pathology.

All steps of construction (providing material, packing, sterilizing etc.) performed by *Asia Jarah Pishro Co. Ltd. (address: No. 29, Khorshid Alley, Daemi St., Fatemi Ave., Tehran, Iran and website:* https://asiajarah.com*)*. Every novel single-drape cover had 2 Z shape folds in the middle itself and was made up of a clear medical grade polyethylene film in size 180 × 90 cm.

**Fig. 2:**
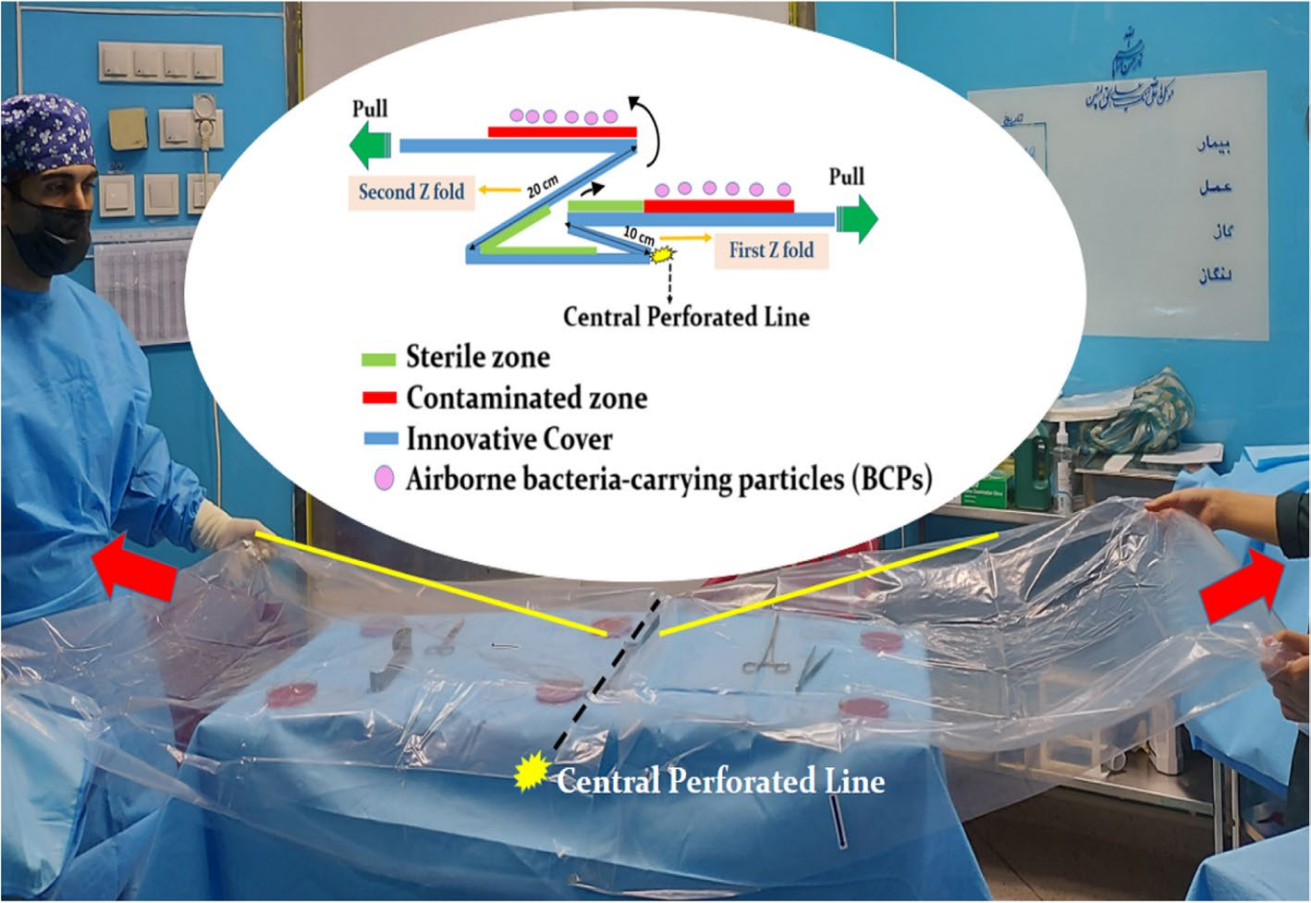
The novel single-drape cover with medical grade polyethylene texture

The novel single-drape cover was designed in a way to easily separate the middle of cover by central perforated line when, no longer needed it. Hence, it may decrease the potential for airborne bacterial contamination of the PSBTs during application and removal compared to using other methods. The central perforated line placed beneath 2 Z shape folds (upper fold were 20 cm and lower fold 10 cm length). Drawing back the cover’s 2 Z folds from the central perforated line may prevent falling the ABCPs that settle on roof of cover onto PSBTs when, two person tore the novel single-drape cover along the central perforated line to remove it. In sum, the aim of design this cover was to protect the PSBTs and to help maintaining sterility during removal it (Fig. 2).

### Static testing

For static testing, at each time point (15, 30, 45, 60, 120, 180, 240 min and 24 h) 3 back Tables (45 total, at each time-point 3 PSBTs were tested 3 occasions for 3 groups). 3 back tables were prepared with draping and putting many sterile surgical instruments on their surface and subsequently, covered by the standard two-drape method or the novel single-drape cover except of group 1.

To evaluate the beginning of plates contamination on PSBTs in time-points 15, 30 and 45 min, the plates were put on the same back tables prepared for evaluating contamination in time points 60 min in Group 1. Hence, plates were collected after 15, 30 and 45 min from PSBT in Group1. Each of the 3 back tables was located around the circumference of the sterile the OR a minimum of 60 cm below the OR bed a minimum of 95 cm distance from the walls, and at the borders of the ceiling diffuser arrays (Fig. 1. D). The surface of back tables was prepared by scrub person with a disposable nonwoven sterile drape. The package of surgical instrument trays was opened by circulator person and a few sterile instruments were put on the PSBTs by scrub person. Then, agar plates (four 5% *Sheep blood ager* and two *MacConkey* agar plates) were aseptically placed on PSBTs beside of surgical instruments by the circulator person.

Then, in Group 1, agar plates (four 5% sheep blood ager and two *MacConkey* agar plates) were placed on the PSBTs (6 plates on the PSBT per each time-point 15, 30, 45, 60, 120, 180, 240 min and 24 h; 72 plates in total at time-points 15 to 60 min) beside of surgical instruments by the scrub person.

In Group 2, after the 6 plates aseptically were placed on the PSBTs, the first drape was placed horizontally and with the cuff at the halfway point over the top of the instruments and then, the second drape is placed from the opposite side and completely covers the cuff of the first drape by scrub person. After draping, 6 plates else were placed on top of the drapes (12 plates on PSBT per each time point).

In Group 3 similar to group 2, after plates were placed on PSBT, the novel single-drape cover was aseptically placed over the top of the instruments and the agar plates. Next, 6 plates else were placed on top of the novel single-drape cover. Finally, in all groups, plates at each time point ware aseptically collected. In Group 2 and 3 when covers were removed, the plates under were collected.

### Dynamic testing

All actions performed in static testing repeated again for dynamic testing, with the difference that PSBTs were evaluated in the OR with mock surgical procedure at the different time points (15, 30, 45, 60, 120, 180, and 240 min; except time point 24 hour because, it was evaluated in static condition) (Fig. 1. E).

### Mock surgical procedure and personnel

In order to provide consistent current of the mock surgical procedures and to ensure unbiased repeatability, a detailed, timed process was developed and showed on a laptop monitor within the OR. This script defined the physical actions (roles play) for each of the research team members to perform in 4-minute increments during the 15–240 min mock surgical procedures to simulate actual OR conditions. The mock surgical procedures were designed by the authors, consulting with 6 surgeons, and based on similar studies [7, 16, 18–21] and next the validity of different steps the Persian version of the scripts was confirmed by 10 faculty members (excerpt of 60-minute script is shown in Table 1).

**Table 1:** Piece of mock surgical procedure script that should be performed in time point 60 min. The research team followed a detailed script that provided specific direction of movements and actions every 4 minutes to closely simulate an actual surgical procedure

Mock Surgical Procedure	
**0–4 min**	1) SP performed preoperative surgical hand scrub and the CP performs only hand disinfection. 2) CP opens a disposable sterile surgical gowns and drapes package and a surgical instrument tray on a separate table from 3 back Tables. 3) SP dons surgical gown and gloves before establishing the sterile field. 4) CP places ESU and suction machine close to sterile field. 5) To prepare sterile conditions*,* SP drapes the unsterile surfaces of 3 back tables and Mayo stand by disposable nonwoven sterile drapes and then place the 4–6 sterile surgical instrument on each 3 back tables in line.
**1 min**	**Decreased surgical Activity in sterile field (Resting position)**
**5–8 min**	1) SP places instruments on the Mayo stand for passing them to the surgeon during mock surgical procedure. 2) To prepare sterile filed, SP covers total surface of OR bed by 2 sterile sheet. 3) After scrubbing, the surgeon and surgical assistance wear gown and gloves for entering the sterile filed. 4) The CP wears sterile gloves and placed 6 agar plates (4 sheep blood agar and 2 MacConkey agar plates) on each 3 back table, 1 in each corner and 2 in the middle of the table (in Group 1, we placed 18 extra agar plates on back tables that every 6 plates were related to each of time points 15, 30 and 45 min) and removed the lids. 5) Nurse anesthetist stands above the OR bed. Body movement: walking to a cabinet and back again 6) Entering and leaving CP for obtaining additional supplies and equipment or surgical instruments (2 times).
**1 min**	**Decreased surgical activity in sterile field (Resting position)**
**9–12 min**	1) After plates were placed on back tables in group 2 and 3, SP drapes the PSBT by the two-drape method and the novel single-drape cover, respectively. Next, CP puts 6 agar plates else on top of the drapes. 2) CP measures and records OR temperature and humidity for every experiment. 3) After covering total surface of unsterile OR bed, CP places a sausage in center of it. 4) Prep and draping the patient (a sausage) by SP 5) Entering and leaving the CP (1 time). 6) The CP opens the Bovie pencil on a sterile field 7) Passing instruments to the surgeon during mock surgical procedure
**1 min**	**Decreased surgical activity in sterile field (Resting position)**
**13–16 min**	1) Surgeon places the sterile active electrode tip against the sausage (Mock patient) to generate particulate tissue matter (10 second per 1 min). 2) Hand and arm movement by surgeon: continuous random finger motions close to the mock patient (1 min) 3) Hand and arm movement: Passing instruments to the surgeon during mock surgical procedure (repeated) 4) Entering and leaving the CP (1 time). 5) CP moves in OR for monitoring the sterile field and the members of the sterile team. 6) Hand and arm movement: The surgical instruments are moved between surgical field and mayo stand
**1 min**	**Decreased surgical Activity in sterile field (Resting position)**

**SP:** Scrub person; **ESU:** Electrosurgery unit; **CP:** Circulator person; OR: Operating room

The script is simulated the actual steps undertaken by research team members and includes gowning and gloving, personnel entering and leaving the room (15 times per experiment), putting typical sterile surgical instruments on the mayo stand after covering it with sterile drape, passing instruments, use of electrocautery on an uncooked sausage (mock patient) to create particulate tissue matter and etc. (Table 1). Intraoperatively, the scrub person used sterile instruments on Mayo stand and did not touch the instruments on the PSBTs so as not to disturb the covers.

The study team included a surgeon; a surgeon assistance; 3 OR personnel (a scrub person, a circulator person and a nurse anesthetist) and the researcher. Mock surgical procedures were performed by the same team. Preoperatively, the members of mock surgical team wore standard hospital-issued, caps, facemasks, clean surgical attire, and shoe covers and after surgical hand scrub, they wore sterile gowns and surgical gloves. During preparation, procedure and data collection within the OR, the circulator person just put on cap, facemask, clean surgical attire, and shoe covers. He opened aseptically sterile goods on back tables and also provided all needs of team for mock surgical procedures.

### Microbiology

All plates were analyzed by one team’s microbiologist and quantified as colony forming units per plate (CFU/plate). In the present study, the way of passive measuring CFUs was used by settle plate method instead of other passive one such as swab or active method such as impactor air sampler [12, 20, 22, 23] because the risk of failing to catch the bacteria on the swab was considered greater compared to the settle plates [20]. airborne bacterial contamination of the PSBTs was then passively measured by 6 agar plates placed on each them.

The ager plates (9 cm in diameter) were purchased from a Clinical Microbiology Laboratory, Kashan, Iran. The laboratory was performed to control quality of every batch of them before to delivery. The collected 5% Sheep blood agar and *MacConkey* agar plates were incubated at 36 °C under aerobic conditions. Those were evaluated (e.g. staining, microscopic, and biochemical properties of the microorganisms) by the expert microbiologist at the clinical Microbiology Laboratory, Kashan University Hospital.

After 24–48 hour of incubation, bacterial growth was determined quantitatively by counting CFUs/plate and classified according to species. The Standard Antimicrobial susceptibility testing (AST) for *staphylococcus* and *Enterobacter* species was performed by the Kirby-Bauer disk diffusion test (6.4 mm; *Padtan-TEB Co., Tehran, Iran*) for *Azithromycin* (10 mg), *Rifampin* (5 mg), *clindamycin* (2 mg), gentamicin (10 mg), Co. *trimoxazole/Sulfamethoxazole* (1.25 mg), *Ceftazidime* (30 mg), *Amikacin* (30 mg), *Meropenem* (10 mg), *Cefepime* (30 mg), *Piperacillin* (10 mg), *Ceftriaxone* (30 mg), *Ampicillin* (10 mg), and *Cefotaxime* (30 mg) with a 0.5 McFarland bacterial suspension in 0.85% NaCl on *Mueller-Hinton* agar plates (*Merck KGaA, Darmstadt, Germany*).

After 16–20 hour of incubation at 36 °C, the inhibition zone diameters were measured and each isolation was evaluated according to a standard protocol (The Clinical and Laboratory Standards Institute [CLSI] on AST [https://clsi.org]). Considered multidrug resistant (MDR), If detected bacteria were resistant to at least three of the antibiotic groups tested.

### Statistical analysis

Data analysis was performed via descriptive statistics (frequency [Percentage] and Mean [SD]) and statistical tests (*Kruskal–Wallis* [KW] test followed by post hoc *Mann-Whitney* U-test and *Bonferroni* correction test) using IBM SPSS version 22.0 (IBM Corp., Armonk, NY, USA). Statistical significance was considered when *P*-value < 0.05.

## Results

In the present study 2361 bacterial colony with different bacterial species were found on 442 (48.1%) blood agar plates from 918 plates, with the most common being *coagulase-negative Staphylococcus* (n:1801 [46.6%]) followed by *Corynebacterium diphtheria* (n: 226 [14.2%]) and *Bacillus* spp. (n: 207 [13.9%]). Of 2361 bacterial colony isolated, 4 pathogenic colonies (*Staphylococcus aureus* [n:2]; *Enterobacter species* [n:2]) that in terms of AST were tested. Both isolated *Staphylococcus aureus* colony were resistant to Azithromycin and Clindamycin antibiotic disc and both isolated *Enterobacter species* were resistant to Ampicillin.

Covering the PSBTs during static periods resulted in a lower bioburden of ABCPs underneath the covers compared to on top of the covers at five time points 60, 120, 180, 240 min and 24 hours. Interestingly, there was no significant difference in the bioburden of ABCPs beneath the covers when, Group 2 and 3 at the mentioned time points were directly compared (Table 2). Also, there was no significant difference between the bioburden of ABCPs on top of each of the covers in Group 2,3 and PSBTs no cover in Group 1 at all (Tables 2, 3).

**Table 2:** Comparison of the total mean detected CFUs/Plate in 3 Groups during different time-points of experiment

Experiment Time points	Periods testing	No. of CFUs/plate			*P-value
		No cover	The standard two-drape method (Beneath cover)	The novel single-drape cover (Beneath cover)	
		Mean ± SD	***Mean*** ± SD	Mean ± SD	
**60 min**	Static	1.72 ± 1.64	0.22 ± 0.943	0.22 ± 0.943	< 0.0001
	Dynamic	2.6 ± 2.83	0.33 ± 0.686	0.11 ± 0.323	0.001
**120 min**	Static	2.72 ± 2.65	0.44 ± 0.70	0.11 ± 0.32	< 0.0001
	Dynamic	3.89 ± 5.58	0.28 ± 0.67	0.22 ± 0.55	0.001
**180 min**	Static	3.33 ± 3.10	0.33 ± 0.59	0.17 ± 0.38	0.001
	Dynamic	4.50 ± 4.29	0.61 ± 1.38	0.28 ± 0.67	< 0.0001
**240 min**	Static	5.28 ± 4.56	0.61 ± 0.78	0.17 ± 0.38	.002 0
	Dynamic	6.94 ± 5.64	0.61 ± 0.60	0.39 ± 0.60	003 0
**24 hours**	Static	12.4 ± 10.7	0.72 ± 1.2	0.22 ± 0.43	0.002

*CFUs: *Colony-forming units

* *P* < 0.05 is significant

**Table 3:** Comparison of the total mean detected CFUs/Plate above the cover compared to beneath the cover in Groups 2 and 3 during different time-points of experiment

Experiment Time points	Periods testing	Groups	No. of CFUs/plate		*P-value
			Top cover	Beneath cover	
			Mean ± SD	Mean ± SD	
**60 min**	**Static**	The standard two-drape method	1.98 ± 2.30	0.22 ± 0.94	0.005
		The novel single-drape cover	1.89 ± 1.84	0.11 ± 0.32	0.003
		***P*-value**	0.93	0.60	
	**Dynamic**	The standard two-drape method	2.28 ± 2.89	0.33 ± 0.67	0.031
		The novel single-drape cover	1.78 ± 2.045	0.22 ± 0.43	0.027
		***P*-value**	0.66	0.86	
**120 min**	**Static**	The standard two-drape method	2.28 ± 2.052	0.44 ± 0.70	0.002
		The novel single-drape cover	1.89 ± 1.937	0.11 ± 0.32	0.003
		***P*-value**	0.64	0.56	
	**Dynamic**	The standard two-drape method	2.83 ± 2.53	0.28 ± 0.67	0.005
		The novel single-drape cover	4.00 ± 4.14	0.22 ± 0.55	0.002
		***P*-value**	0.79	0.26	
**180 min**	**Static**	The standard two-drape method	3.50 ± 3.034	0.33 ± 0.59	0.004
		The novel single-drape cover	2.50 ± 2.61	0.17 ± 0 .38	0.002
		***P*-value**	0.64	0.56	
	**Dynamic**	The standard two-drape method	3.4 ± 3.45	0.61 ± 1.38	0.007
		The novel single-drape cover	4.8 ± 4.74	0.28 ± 0.67	0.002
		***P*-value**	0.73	0.60	
**240 min**	**Static**	The standard two-drape method	4.22 ± 4.48	0.61 ± 0.78	.01 0
		The novel single-drape cover	3.56 ± 3.365	0.17 ± 0.38	0.001
		***P*-value**	0.50	0.053	
	**Dynamic**	The standard two-drape method	7.22 ± 6.57	0.61 ± 0.78	013 0
		The novel single-drape cover	7.44 ± 6.0	0.39 ± 0.60	0.004
		***P*-value**	0.96	0.22	
**24 hours**	**Static**	The standard two-drape method	10.8 ± 9.55	0.72 ± 1.2	0.004
		The novel single-drape cover	8.33 ± 7.10	0.22 ± 0.43	< 0.0001
		***P*-value**	0.67	0.29	

*CFUs:* Colony-forming units

* *P* < 0.05 is significant

Similar to the results of static testing, covering PSBTs during dynamic periods resulted in a lower bioburden of ABCPs underneath the covers compared to on top of the covers (Time-points 60, 120, 180 and 240 min) (Table 3), but no such significant difference was seen beneath the covers when, Group 2 and 3 at the mentioned time-points were directly compared (Table 3).

**Fig. 3:**
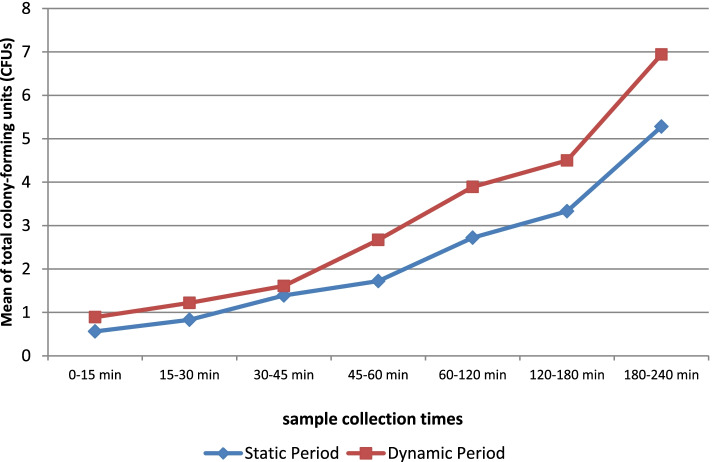
The Trend chart shows an association between increasing time and increasing mean contamination of PSBTs with ABCPs during static and dynamic periods in Group1

Our result showed contamination of PSBTs starts within 0–15 minutes in Group1. There was no significant difference in the starting and mean of contamination of PSBTs with ABCPs during static and dynamic periods in Group1. Also, there was an association between increasing time and increasing mean contamination of PSBTs with ABCPs during static and dynamic periods in Group1 (Table 4, Fig. 3).

**Table 4:** Comparison of the total mean detected CFUs/Plate and the time-dependent airborne bacterial contamination of PSBTs in Group 1 during static and dynamic periods

Experiment Time points	No. of CFUs/plate		*P-value
	Static period	Dynamic period	
	Mean ± SD	Mean ± SD	
**0–15 min**	0.56 ± 0.86	0.89 ± 1.18	0.56
**15–30 min**	0.83 ± 1.34	1.22 ± 1.73	0.52
**30–45 min**	1.39 ± 1.8	1.61 ± 1.94	0.72
**45–60 min**	1.72 ± 1.64	2.67 ± 2.82	0.72
**60–120 min**	2.72 ± 2.65	3.89 ± 5.58	0.88
**120–180 min**	3.33 ± 3.106	4.50 ± 4.287	0.42
**180–240 min**	5.28 ± 4.56	6.94 ± 5.64	0.25

* *P* < 0.05 is significant

Similarly, this association was seen on the top of each of covers in Groups 2 and 3 during static and dynamic periods, but no such association was seen beneath the covers (Table 3).

## Discussion

This study was designed to test the hypothesis that using a novel single-drape cover had more efficiency and safety in decreasing ABCPs settling on the PSBTs during static and dynamic periods than the standard two-drape method.

The results of our study showed no significant difference in the bioburden of ABCPs beneath the covers, when group 2 and 3 at the mentioned time points were directly compared (*P* < 0.05). The use of the standard two-drape method currently recommends by the AORN while, our findings showed there is no preference for using the novel single-drape cover compared to two-drape method. But, due to economic issues may many hospitals may resist to cover PSBTs by two disposable drapes and prefer our transparent and cost effective the novel single-drape cover to a non-transparent cover in the standard two-drape method.

The results of our study showed covering PSBTs in the OR during static and dynamic testing (time-points 60, 120, 180, 240 min and 24 hours) resulted in a significantly decreased ABCPs on the PSBTs (P < 0.05). In line with our findings, *Markel* et al. [12] have shown to covering the PSBTs during static and dynamic OR conditions resulted in a significantly decreased bioburden of ABCPs on the PSBTs in time-points 60 min, 4 and 8 hours (above cover 1, 5.5; IQR, 9.5; beneath cover 1, 0; IQR, 1; *P* < .0001; above cover 2, 14; IQR, 22.5; beneath cover 2, 0; IQR, 0.25; P < .0001).

Also, their study showed no difference in the bioburden of ABCPs beneath the covers, when the sterile plastic and paper covers were directly compared (*P* = 0.1). At 24 hours, their results of study showed no significant difference bioburden of ABCPs above and beneath the cover of PSBTs at 24 hours (above cover, 0; IQR, 0.25; beneath cover, 0; IQR, 0; *P* = 0.1) and it was inconsistent with results of our study (Group 2, *P* = 0.004; Group 3, *P* = 0.0001).

That seems possible reason this discrepancy to be that our study at 24 hour tests were performed in OR, whereas the 24-hour test in *Markel* et al. study was performed in adjacent of an OR, where foot traffic and the possibility of contamination PSBTs were higher.

In line with our findings, *Qvistgaard* et al. [11] indicated that to cover PSBTs properly with at least a single-layer drape before a surgical procedure is good practice but, it is a better option to support them with a double-layer drape. Because, covering with double-layer drape are significantly reduced the CFUs/plate compared to the single-layer drape.

A study by *Wistrand* et al. [20] showed that the uncovered PSBTs had 98 CFUs/plate versus 20 in the covered PSBTs during static periods (*P* < 0.0001). Also, they declared protecting PSBTs from ABCPs with sterile covers enhances the durability of their sterile items up to 24 h.

Our study showed no statistical difference between the bioburden of ABCPs on top of each of the covers in Group 2,3 and PSBTs no cover in Group 1 at all (*P* < 0.05). These results were in line with findings of *Markel* et al *study* [12]. (*P* = 0.19). So, the bacterial bioburden on PSBTs no cover was equivalent to the bioburden on top of each cover in Group 2 and 3, again confirming there was no break or bias in our methodology.

In the present study the most common bacteria detected on the plates were coagulase-negative Staphylococcus (n:1801 [46.6%]). In line with the present study, earlier studies have shown the most common bacteria detected on the contaminated instruments had coagulase-negative Staphylococcus 60.4% (28) and 44% [16]. Because, coagulase-negative Staphylococcus were frequently isolated from air samples obtained throughout the OR, they were recovered from 86% of air samples [24].

The results of our study showed contamination of PSBTs is started within 0–15 minutes in Group1. There was no statistical difference in the start and mean airborne bacterial contamination of PSBTs during static and dynamic periods in Group1(*P* < 0.05).

While, in real surgery conditions, *Uzun* et al. [25] showed bacterial growth on PSBTs started after 30 and 60 min in the uncovered sets (6 of 30 [20%]) and covered groups (2 of 30 [6.7%]), respectively (*P* = 0.024). *Dalstrom* et al. [16] found that no contamination has occur in the covered group while, rate of 30% contamination appeared in group without cover after 4 hours. The results of both studies was different together, and also was inconsistent with results of our study, and perhaps the differences in starting airborne bacterial contamination of the uncovered PSBTs in our study and two studies else, be related to differences in ORs condition, study deign, and ventilation systems. We did not evaluate the beginning of PSBTs contamination at different time-points in the covered groups, because accessing the covered PSBTs would have disturbed the covers that were being assessed for sterility.

There was a correlation between increasing time (15 to 240 min) and increasing mean contamination of PSBTs with ABCPs during static and dynamic periods in Group1. Also, the mentioned association was seen on top of each of the covers in Groups 2 and 3 during static and dynamic periods, but no such association was seen beneath the covers. In line with our findings, *Wistrand* et al. [20] showed there was a positive correlation between increasing time (0 to 24 hours) and number of total CFUs/plate (2 to 30 CFUs/plate) in the group uncovered during static periods, while no such correlation was seen in the covered group (CFUs/plate: 2 to 7). They believed when PSBTs are covered, time has little effect on airborne bacterial contamination.

*Uzun* et al. [25] demonstrated the contamination of uncovered and covered PSBTs increases with over time (uncovered group; contamination rate instrument trays with increasing time was 20% at 30 min vs. 43.4% at 120 min). Hence, covering may serves as a barrier to ABCPs and several research support the covering sterile areas to diminish the potential for contamination in the OR [12, 15, 16]. Although, under normal situation, it is rare that surgical instrument trays open and left unattended in the OR unless short delays in begging of surgery occur. In these circumstance, the PSBTs may realistically be covered for a short period before the begin of the surgery [12, 20]. To decrease the potential for intraoperative contamination., every effort should be employed to reduce the exposure of sterile filed. The use of the standardized disposable innovative covers may minimize the exposure to environmental contaminants. In line with our findings, the study of *Kaska* showed the use of a disposable novel sterile drape (a novel sterile C-arm drape) that maintains the integrity of the sterile field, is reduced SSI risk factors and improved OR efficiency [26]. Therefore, maintaining integrity of the sterile field via a standard innovative sterile drape used to cover sterile and unsterile objects, may potentially reduce SSIs.

### Limitations

We noticed that the present study had two limitations.

First, we realized that a criticism of this study may be why present study did not perform in real operations. Because of health privacy laws and ethical considerations, our experiments were performed during a mock surgical procedure instead of a real operation with patients. However, the conditions of the mock procedure were very close to that of a real surgery; therefore, the results are likely able to be comparable.

Second potential limitation could be not accessing the scrub person to covered PSBTs during the mock surgical procedure. Accessing the PSBTs may have been more realistic, but would have disturbed the covers that were being assessed for PSBTs.

## Conclusions

The present study showed to cover PSBTs is a simple action that effectively reduces contamination over both short and long periods of time. Furthermore, it is suitable and logical practice to cover PSBTs or every sterile fields else properly with sterile cover (whether the novel single-drape cover or the two-drape method) during static and dynamic periods. Because, the contamination of PSBTs increases with time and its rates may be decreased by covering.

Hence, we suggest to use of back table covers during periods of unanticipated delay, increased activity (e.g. during preoperative patient skin antisepsis or patient positioning), procedures with multiple back tables and a sterile field that has been prepared and will not immediately (nonuse).

Our findings demonstrated that there is no preference for using the novel single-drape cover compared to the two-drape method. But due to economic issues many hospitals resist to cover PSBTs by two disposable drapes and prefer our transparent and cost effective the novel single-drape cover to a non-transparent cover in the two-drape method.

As shown in our results, we cannot exclude that there may be a risk of contamination when removing the covers (whether the novel single-drape cover or the two-drape method), but the protective effect is higher compared to the risk of contamination.

Also, covering can be used when, the OR personnel are on call, and hence not present at the OR and unable to promptly prepare sterile items (e.g., trauma, acute fetal distress etc.), and when a variety of surgical instrument trays are simultaneously opened for a surgery but, intraoperatively, are not immediately used (e.g., arthroplasty surgery, scoliosis repair, CABG surgery, etc.) or when increased activity in the OR (e.g., during preoperative patient skin antisepsis or patient positioning, etc.).

## Data Availability

The data analyzed during the current study are available from the corresponding author on reasonable request.

## Abbreviations

AORN: The Association of periOperative Registered Nurses
ABCPs: Airborne bacteria-carrying particles
APs: Airborne particles
CP: Circulator person
ESU: Electrosurgery unit
OR: Operating room
PSBTs: Prepared sterile back tables
SSIs: Surgical site infections
SP: Scrub person

## References

[1] Persson M (2019). Airborne contamination and surgical site infection: could a thirty-year-old idea help solve the problem?. Med Hypotheses.

[2] Chauveaux D (2015). Preventing surgical-site infections: Measures other than antibiotics. Orthop Traumatol Surg Res.

[3] Parvizi J, Barnes S, Shohat N, Edmiston CE (2017). Environment of care: is it time to reassess microbial contamination of the operating room air as a risk factor for surgical site infection in total joint arthroplasty?. Am J Infect Control.

[4] Talon D, Schoenleber T, Bertrand X, Vichard P (2006). Performances of different types of airflow system in operating theatre. Ann Chir.

[5] Howorth FH (1985). Prevention of airborne infection during surgery. Lancet..

[6] Brown J, Doloresco F, Mylotte JM (2009). “Never events”: not every hospital-acquired infection is preventable. Clin Infect Dis.

[7] Gormley T, Markel TA, Jones HW, Wagner J, Greeley D, Clarke JH (2017). Methodology for analyzing environmental quality indicators in a dynamic operating room environment. Am J Infect Control.

[8] Whyte W, Hodgson R, Tinkler J (1982). The importance of airborne bacterial contamination of wounds. J Hosp Infect.

[9] Pasquarella C, Pitzurra O, Herren T, Poletti L, Savino A (2003). Lack of influence of body exhaust gowns on aerobic bacterial surface counts in a mixed-ventilation operating theatre. A study of 62 hip arthroplasties. J Hosp Infect.

[10] Sunagawa S, Koseki H, Noguchi C, Yonekura A, Matsumura U, Watanabe K (2020). Airborne particle dispersion around the feet of surgical staff while walking in and out of a bio-clean operating theatre. J Hosp Infect.

[11] Qvistgaard M, Almerud-Österberg S, Lovebo J. Covering surgical instruments with single- or double-layer drape pending surgery: an experimental study in a perioperative setting. J Infect Prev. 2020;1757177420973753. 10.1177/1757177420973753. 10.1177/1757177420973753 PMC8113674 34234845

[12] Markel TA, Gormley T, Greeley D, Ostojic J, Wagner J (2018). Covering the instrument table decreases bacterial bioburden: an evaluation of environmental quality indicators. Am J Infect Control.

[13] Van Wicklin SA (2018). Are knowledge and attitudes of perioperative registered nurses associated with the practices of covering and monitoring sterile tables?. Perioper Care Oper Room Manag.

[14] Fallis A (2013). AST standards of practice for creating the sterile field. J Chem Inf Model.

[15] Chosky S, Modha D, Taylor G (1996). Optimisation of ultraclean air: the role of instrument preparation. J Bone Joint Surg Br.

[16] Dalstrom DJ, Venkatarayappa I, Manternach AL, Palcic MS, Heyse BA, Prayson MJ. Time‑dependent contamination of opened sterile operating‑room trays. JBJS. 2008;90(5):1022-5. 10.2106/JBJS.G.00689. 10.2106/JBJS.G.00689 18451394

[17] Dharan S, Pittet D (2002). Environmental controls in operating theatres. J Hosp Infect.

[18] Gormley T, Markel TA, Jones H, Greeley D, Ostojic J, Clarke JH (2017). Cost-benefit analysis of different air change rates in an operating room environment. Am J Infect Control.

[19] Qvistgaard M, Lovebo J, Almerud-Österberg S (2019). Intraoperative prevention of surgical site infections as experienced by operating room nurses. Int J Qual Stud Health Well-being.

[20] Wistrand C, Söderquist B, Sundqvist AS (2021). Time-dependent bacterial air contamination of sterile fields in a controlled operating room environment: an experimental intervention study. J Hosp Infect.

[21] Annaqeeb MK, Zhang Y, Dziedzic JW, Xue K, Pedersen C, Stenstad LI (2021). Influence of surgical team activity on airborne bacterial distribution in the operating room with a mixing ventilation system: a case study at St. Olavs Hospital J Hosp Infect.

[22] Fu Shaw L, Chen IH, Chen CS, Wu HH, Lai LS, Chen YY (2018). Factors influencing microbial colonies in the air of operating rooms. BMC Infect Dis.

[23] Matinyi S, Enoch M, Akia D, Byaruhanga V, Masereka E, Ekeu I (2018). Contamination of microbial pathogens and their antimicrobial pattern in operating theatres of peri-urban eastern Uganda: a cross-sectional study. BMC Infect Dis.

[24] Edmiston CE, Seabrook GR, Cambria RA, Brown KR, Lewis BD, Sommers JR (2005). Molecular epidemiology of microbial contamination in the operating room environment: is there a risk for infection?. J Surg.

[25] Uzun E, Misir A, Ozcamdalli M, Kizkapan EE, Cirakli A, Calgin MK (2020). Time-dependent surgical instrument contamination begins earlier in the uncovered table than in the covered table. Knee Surg Sports Traumatol Arthrosc.

[26] Kaska SC (2010). A standardized and safe method of sterile field maintenance during intra-operative horizontal plane fluoroscopy. Patient Saf Surg.

